# Prediction of the Chemical Context for Buchwald‐Hartwig Coupling Reactions

**DOI:** 10.1002/minf.202100294

**Published:** 2022-02-22

**Authors:** Samuel Genheden, Agnes Mårdh, Gustav Lahti, Ola Engkvist, Simon Olsson, Thierry Kogej

**Affiliations:** ^1^ Molecular AI Discovery Sciences R&D AstraZeneca Gothenburg SE-431 83 Mölndal Sweden; ^2^ Chalmers University of Technology Department of Computer Science and Engineering Rännvägen 6 41258 Göteborg Sweden

**Keywords:** context prediction, condition prediction, Buchwald-Hartwig coupling reactions, CASP

## Abstract

We present machine learning models for predicting the chemical context for Buchwald‐Hartwig coupling reactions, i. e., what chemicals to add to the reactants to give a productive reaction. Using reaction data from in‐house electronic lab notebooks, we train two models: one based on single‐label data and one based on multi‐label data. Both models show excellent top‐3 accuracy of approximately 90 %, which suggests strong predictivity. Furthermore, there seems to be an advantage of including multi‐label data because the multi‐label model shows higher accuracy and better sensitivity for the individual contexts than the single‐label model. Although the models are performant, we also show that such models need to be re‐trained periodically as there is a strong temporal characteristic to the usage of different contexts. Therefore, a model trained on historical data will decrease in usefulness with time as newer and better contexts emerge and replace older ones. We hypothesize that such significant transitions in the context‐usage will likely affect any model predicting chemical contexts trained on historical data. Consequently, training context prediction models warrants careful planning of what data is used for training and how often the model needs to be re‐trained.

## Graphical abstract

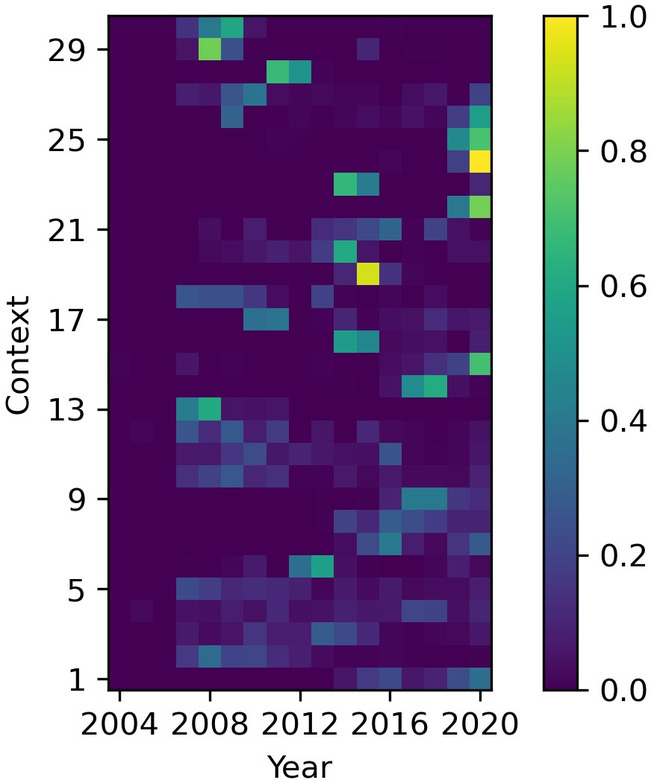

## 1 Introduction

The use of computer‐aided synthesis planning (CASP) started several decades ago but has gained a renewed interest recently with recent advances in machine learning.[^1^, ^2^, ^3^] CASP is beneficial for both medicinal and process chemists as the tools provide suggestions on how to synthesize novel compounds and guide the optimization of reaction conditions. Coupling powerful machine learning models to large databases of reactions such as Reaxys,^[4]^ CAS,^[5]^ Pistachio^[6]^ or internal corporate Electronic Laboratory Notebooks (ELNs), has the potential to transform the way chemists approach synthesis.

Reaction outcomes are susceptible to minute changes in the conditions of the reaction, e. g. temperature, pressure, solvent, catalyst. Consequently, getting the right set of conditions, i. e., the reaction context, is critical in organic synthesis. Nonetheless, condition prediction has received relatively little attention compared to other CASP research areas, such as forward reaction prediction and retrosynthesis analysis. Although extensive efforts have sought to formalize the rules for condition optimization, initial conditions selection remains dependent on human intuition. Previously reported *in silico* methods typically only apply for specific reaction classes or parts of the reaction condition, e. g., solvent or catalyst.[^7^, ^8^, ^9^] Gao et al., on the other hand, trained a neural network model to predict the chemical species (catalysts, solvents, and reagents) as well as the temperature most suitable for any given reaction, using 10 million reactions from Reaxys.^[10]^ The authors found a close match to the recorded context, within the top‐10 suggestions in about 70 % of the time, whereas the accuracy for individual species was much higher. Ryou *et al*. proposed a related model based on a graph neural network that predicts conditions for four reaction classes.^[11]^ Their results are impressive for individual reaction species, but less competitive in the combination of the conditions, obtaining a top‐3 accuracy of at most 70 %. Maser *et al*. later refined the work of Ryou *et al*. to exploit the multi‐label nature of the data, i. e., each reaction can have more than one recorded context.^[12]^ This work is, to our knowledge, the only multi‐label approach reported for condition prediction. The advantage of including multi‐label data is that the model may assign probabilities to different contexts rather than just predicting one. This property enables the model to be used in library generation. In such a scenario, it will sometimes be necessary to select a context that is not optimal for one particular reaction, but rather optimal for a set of reactions.

One problem with the models discussed above is that the accuracy for the entire context is low compared to the accuracy for the individual species, a natural effect of modeling the different species individually. To the best of our knowledge, no one has developed a model that jointly predicts the *chemical context* of a reaction, i. e., a fixed set composed of a catalyst, a solvent, and reagents, which are treated as a single label during modeling. Although the methods mentioned above attempt to couple predictions of individual conditions in various ways,[^10^, ^12^] the predictions of different conditions are essentially independent. Instead, modeling the full chemical context would have the advantage of circumventing the error propagation problem plaguing earlier proposed methods.[^10^, ^12^] However, because different reaction classes use different sets of chemical contexts, creating a general‐purpose model applicable to any reaction is challenging. Furthermore, predicting chemical contexts is limited by the combinations of conditions available in the training dataset thereby naturally limiting the scope of the model and hinder the prediction of novel contexts. On the other hand, a model predicting known sets of conditions avoids predicting combinations of chemical species that are chemically incompatible with one and another.

**Figure 1:**
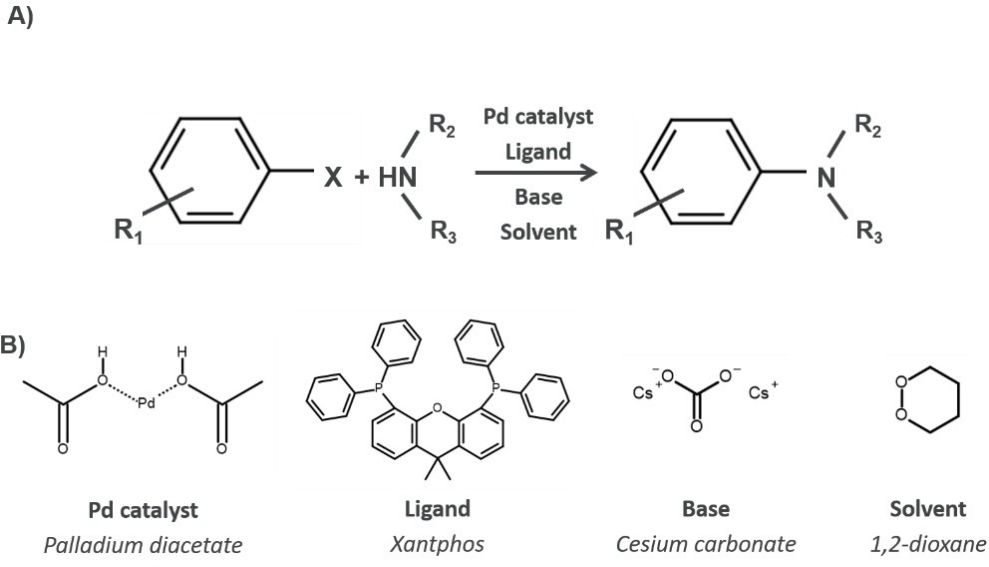
Illustration of A) a generic Buchwald‐Hartwig coupling reaction and B) an example of a chemical context for such a reaction, showing an example of a palladium catalyst, a ligand, a base and a solvent.

Based on these observations and inferences, we decided to create a model to predict chemical contexts for Buchwald‐Hartwig coupling reactions (see Figure 1). Such reactions are ubiquitous in medicinal chemistry,[^13^, ^14^] and, therefore, a good candidate for a prospective study such as this. Furthermore, we compare models trained on single‐label and multi‐label data. Finally, as there is intense research focus on finding new set of conditions for Buchwald‐Hartwig coupling reactions, we will analyze the time‐dependency of the model performance. This is important as our models are, as in the studies discussed above,[^10^, ^12^] based on historical data, i. e. we are attempting to predict context relevant for medicinal chemistry projects today using data from the past. If the historical data is not representative of future preferred reaction conditions, the models may become irrelevant over time.

## 2 Methods

There are two models of primary interest: a single‐label model trained on the highest yielding context for a reaction and a multi‐label model trained on all available data. Both models are feed‐forward neural networks that use reaction fingerprints as inputs and give a chemical contexts as output, i. e., a combination of chemicals to be added to the reactants (see exact definition below).

**Data processing**. We trained the models on reaction data recorded on a subset of the in‐house ELNs covering compounds synthesized between 2004–2020. At the time of data extraction, there were 11,210 recorded Buchwald‐Hartwig reaction variations for single‐product reactions with a yield greater or equal to 20 %. A reaction variation is a recorded reaction with a specific context, whereas a reaction in the database only specifies the reactants and products. The arbitrary limit of 20 % yield is a reasonable compromise between considering the reaction successful enough in a medicinal chemistry context and retaining enough data for modeling. For modeling, we reduced the dataset by only keeping reaction variations that use any of the 30 most common chemical contexts, giving 6,291 reactions.

We featurized the reaction Simplified Molecular‐Input Line‐Entry System (SMILES) into reaction fingerprints by subtracting the sum of the reactant fingerprints from the product fingerprint, i. e. a difference fingerprint.^[15]^ The reactants and product fingerprint consist of a 512‐bit extended connectivity fingerprints with a radius of 3,^[16]^ concatenated to a 512‐bit RDKit fingerprints with a maximum path length of 7.^[17]^ Other featurization schemes of chemical reactions have been suggested recently,[^18^, ^19^] but their use is outside the scope of our study.

Because we are working with Buchwald‐Hartwig reactions, we constructed chemical contexts based on chemicals categorized as catalyst, pre‐catalyst, ligand, base, solvent, or reagent (see Figure 1 for an example). We identified pre‐catalysts and catalysts from a curated list or flagged any chemical containing Pd or Pt elements. We identified solvents using the assignment in the original ELN record. We used atom mappings from the Biovia software to identify reagents as molecules not contributing to the product.^[20]^ Using a curated list of bases and ligands, we assigned these from the list of reagents. We included a “reagent” category since not all chemicals fall into either of these categories. We kept only reaction variations using one of the 30 most common contexts for our model training, all other variations were discarded. For training the single‐label model, we kept only the variant with the highest yield for each reaction if the same reaction occurred multiple times. For training the multi‐label model, we kept only unique reactions, based on the reaction fingerprint and a context identifier. Both the models had their chemical contexts converted to one‐hot vectors. We construct the multi‐hot vectors for training the multi‐label model by merging one‐hot vectors using a bitwise‐or operation. The single‐label data were weighted by the yield, whereas for the multi‐label data no weighting was applied because of the technical complexity of including the yield in the output vector. A summary of the data‐processing is available in Figure S1.

**Model training**. We use the Optuna package for hyper‐parameters optimization,^[21]^ limiting the search space for the single‐ and multi‐label models to the values shown in Table 1. We train the two models using different metrics. For the single‐label model we use the categorical accuracy^[22]^ whereas for the multi‐label model we use the Label Ranking Average Precision (LRAP) score as implemented in Scikit‐Learn^[22]^ (see below). The data was split into a training set consisting of 80 % of the data, and a validation set consisting of 20 % of the data. The training set was used in the hyper‐parameter optimization together with a five‐fold cross validation. The validation set was used to evaluate the convergence and performance of the model with the optimized hyper‐parameters.

**Table 1:** Hyper‐parameter space used for training the two models.

	multi‐label	single‐label
Batch size	32 or 64	32 or 64
Number of epochs	10, 15, or 20	10 or 15
Hidden size	2^7^, 2^8^, or 2^9^	2^7^, 2^8^, 2^9^, 2^10^, or 2^11^
Number of hidden layers	1, 2, or 3	1, 2, or 3
Learning rate	Between 10^−5^ and 5*10^−3^	Between 10^−5^ and 5*10^−3^
Dropout rate	Between 0 and 0.9	Between 0 and 0.9

We arrive at the following optimized architectures and training schedules:

- **Single‐label model**: one hidden layer with 1024 nodes and it has a dropout layer with a dropout‐rate of 0.60 between each layer. It uses ReLU as its activation layer between layers, except for the final layer which uses a softmax activation. The single‐label model was trained using categorical cross‐entropy loss, and Adam optimizer^[23]^ with a learning rate of 6.1*10^−4^. It was trained for 10 epochs using a batch size of 64.
- **Multi‐label model**: one hidden layer with 512 nodes and it has a dropout layer with a dropout‐rate of 0.64 between each layer. It uses ReLU as its activation layer between layers, except for the final layer which uses a softmax function. The multi‐label model was trained using binary cross‐entropy loss, and Adam optimizer with a learning rate of 9.2*10^−4^. It was trained for 20 epochs with a batch size of 64.

We ran three independent training runs to obtain rough uncertainty estimates of the models with the optimal hyper‐parameters.

**Multi‐label metrics**. We evaluate the multi‐label model with two metrics. The LRAP score measures how well the model ranks its ground‐truths by calculating for each ground‐truth how large fraction of the labels with an equal or greater score are also ground‐truths. The Jaccard score^[24]^ measures how well the model predicts the ground‐truths as positive and ground‐falsehoods as not positive. This is calculated by dividing the true positives by the false negatives, true positives, and false positives.

## 3 Results and Discussions

**Figure 2:**
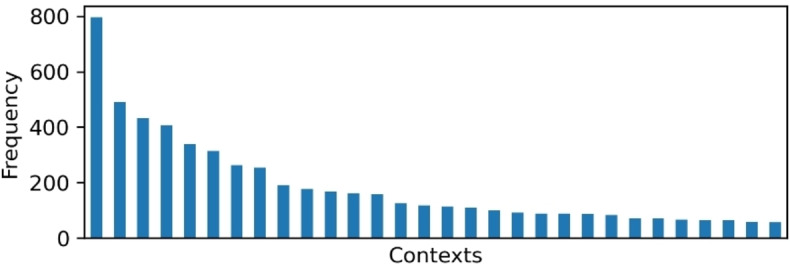
the frequency of the 30 most common contexts for Buchwald‐Hartwig in the dataset used for modeling.

**The dataset is imbalanced and has limited multilabel character**. We first sought to analyze the dataset used for training, which consists of reaction data from the in‐house ELNs. There are 11,210 recorded Buchwald‐Hartwig reactions with a yield greater or equal to 20 %. Out of the 1,000 contexts in the dataset, we only keep the 30 most common contexts, as it is likely that these would be of interest to the chemists. It also follows similar cut‐offs used previously in the literature.[^10^, ^12^] We observe a clear imbalance in the context usage distribution (Figure 2), where the most popular context is used for almost 800 reactions and the less common contexts are only used for about 100 reactions or less. The mean of the imbalance ratios per label is approximately 7.0 and the variance approximately 0.6, and according to the criteria of Charte *et al*. the dataset is considered imbalanced.^[25]^ Similar imbalances have been previously noted in the literature.[^10^, ^12^]

Label cardinality and density measure the degree of multi‐label character of the dataset. The label cardinality is the average number of labels per sample, and the label density is the label cardinality divided by the number of labels. For the current dataset, the label cardinality is 1.01 and the label density 0.033, which shows that most of the data is single‐label and the vectors with the true contexts will be sparse. 86 % of the reactions have only one variation, i. e., more than one recorded context, 11 % have two variations, and 3 % have three or more variations. These statistics underline the sparsity of the dataset used in our study.

**Figure 3:**
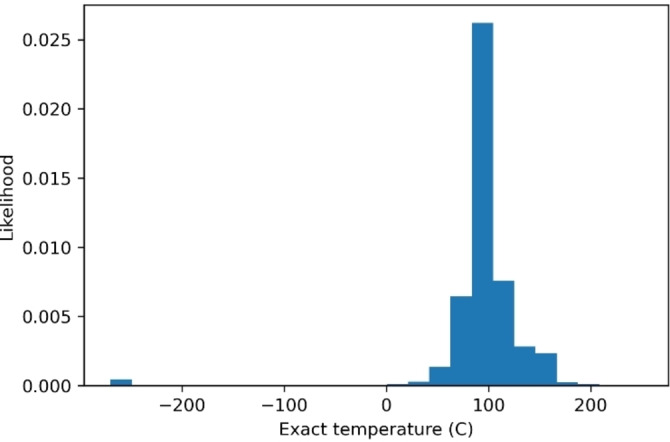
Distribution of temperatures in the dataset. The likelihood is taken as the frequency divided by the total number of data points. There are clearly some incorrect data points with recorded temperatures below −200 degrees Celsius.

**Temperature is excluded from the model**. We next sought to train the models to predict chemical contexts, i. e., a combination of (pre‐)catalyst, ligand, base, and solvent. In contrast to earlier approaches, we chose to exclude temperature in the prediction. As discussed above, our data are sparse and only a few variants are present for each reaction. Some of the contexts infrequently appear in the dataset. One further reason to exclude temperature during modeling is the sparsity of temperature data itself. In particular, for 16.0 % of reactions the temperature is missing, and for 0.2 %, the reported temperature is a range (rather than a single value). In order to incorporate temperatures into modeling, data points would require conversion into a single real number. Previous modeling efforts have replaced missing temperatures with ambient temperature^[11]^ and any corresponding ranges with the midpoint of the range.^[10]^ Interestingly, Gao *et al*. modeled the temperature as a continuous variable with a regression model,^[10]^ whereas Ryou *et al*. and Maser *et al*. modeled it as a discrete variable using binning for the two reaction classes where the temperature data were abundant.[^11^, ^12^] One final reason for excluding temperature is the actual distribution of the temperatures, depicted in Figure 3, since a majority of the recorded temperatures are shown to be around 100 degrees Celsius, a typical temperature for Buchwald‐Hartwig coupling reactions.^[13]^ This narrow distribution of the temperatures further indicates the unnecessary need to precisely predict the temperature for a Buchwald‐Hartwig reaction.

**Figure 4:**
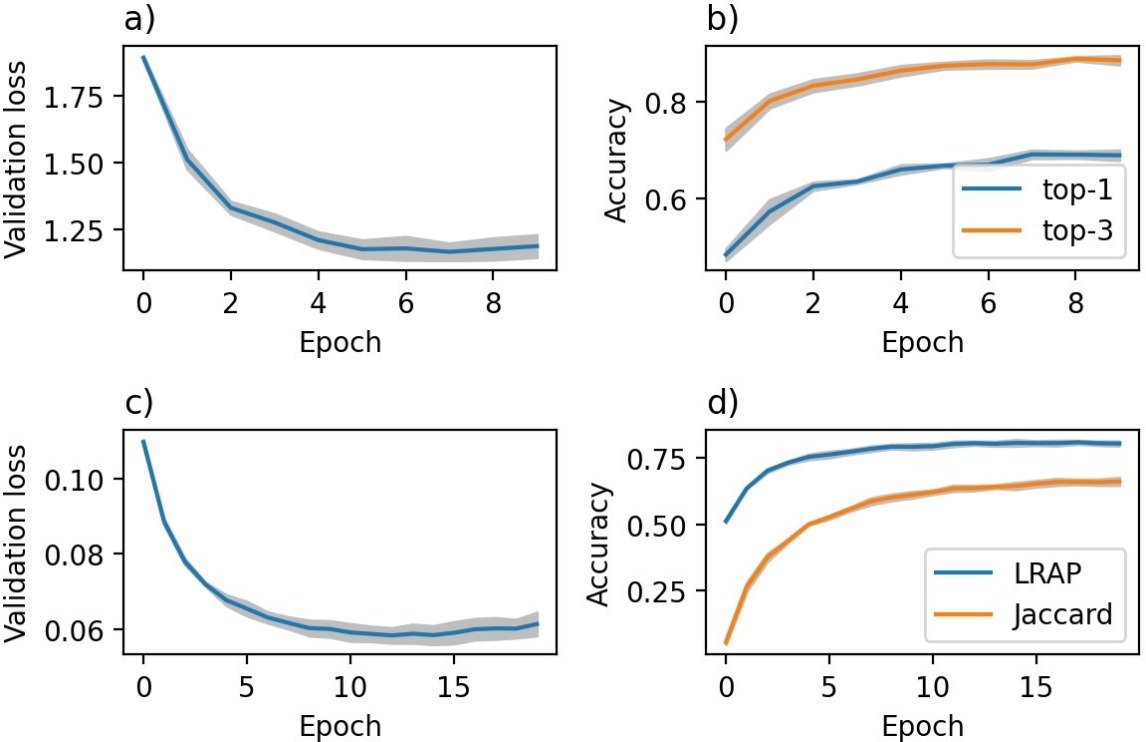
Validation loss and accuracy. a), and b) the single‐label model. c) and d) the multi‐label model. The shaded area is the standard deviation over three independent runs.

**Both single‐label and multi‐label models are performant**. We next sought to analyze the behavioral characteristics of the learning curves to ensure models are trained appropriately. Figure 4a shows the loss as a function of the training epoch and suggests that training has converged. In Figure 4b, we can see that the model achieves a top‐1 accuracy of ∼0.69 and a top‐3 accuracy of ∼0.89. These accuracy scores indicate a well‐performing model, which most often predicts the recorded context highest. The top‐3 accuracy of ∼0.89 is significantly higher than the top‐3 accuracy of approximately ∼0.57 for an exact match of the recorded context presented by Gao *et al*.^[10]^ These results indicate that predicting the conditions as sets, i. e. as contexts, instead of individually provides higher accuracy for predicting the recorded context. A model that suggests the most popular of our context every time gives top‐1 and top‐3 of ∼0.14 and ∼0.18, respectively, which also highlights the single‐label model‘s predictive power.

Similarly, Figure 4C displays the loss curve for the multi‐label model and shows that the loss has stabilized at the end of training. The model has an LRAP score of about 0.81 and a Jaccard score of about 0.68. The high LRAP score suggests that the ranking of the predictions is often correct, meaning that the ground‐truth labels are on average ranked higher than unassociated labels. However, a lower Jaccard score indicates that some predictions are incorrect compared to the ground truth. The label cardinality of the predicted contexts is about 1.0, similar to the label cardinality of the test data. It means that the model reproduces the low label cardinality found in the training data. Thus, the predictions are on average single‐label. It is likely that if the label cardinality of the training data had been higher, the label cardinality of the predictions would be higher as well. A model that suggests the three most popular contexts in order of popularity gives a top‐1 and top3 of ∼0.13 and ∼0.30, respectively, again showing the predictive power of the multi‐label model.

**Figure 5:**
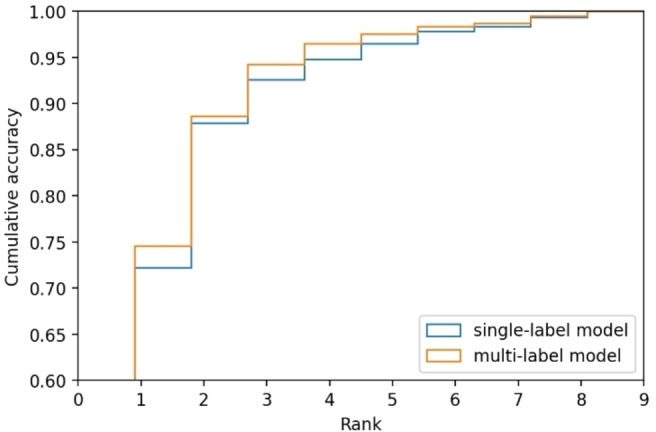
Cumulative accuracy for different ranks. For single‐label model it corresponds to top‐n accuracy, for multi‐label model it corresponds to rank of the lowest scored ground‐truth divided by the number of ground‐truths. Only the first training run is considered in the graph.

**The multi‐label model is marginally more performant than the single‐label model**. Comparing single‐label and multi‐label models is intrinsically non‐trivial, as they are not solving the same task. We compare the models by looking at how they rank the ground truth in their predictions in Figure 5. For the single‐label model, it is the top‐*k* accuracy, which means that if the ground‐truth is predicted with the *k*:th highest score it gets rank *k*. The multi‐label model is the rank of the lowest scored ground‐truth divided by the number of ground‐truths. When there is only one true label, which is the case for most of the data, this calculation will be the same as top‐*k*. More true labels measure how many of the predicted labels that are ranked equal or higher than all true labels are true labels. The performance is similar between the models, although the multi‐label top‐1 accuracy is significantly higher. However, it is difficult to conclude whether this difference in accuracy would make a practical difference. The top‐3 is above 0.9 for both models, which indicates that it would be easy for a chemist to find a good suggestion.

**Figure 6:**
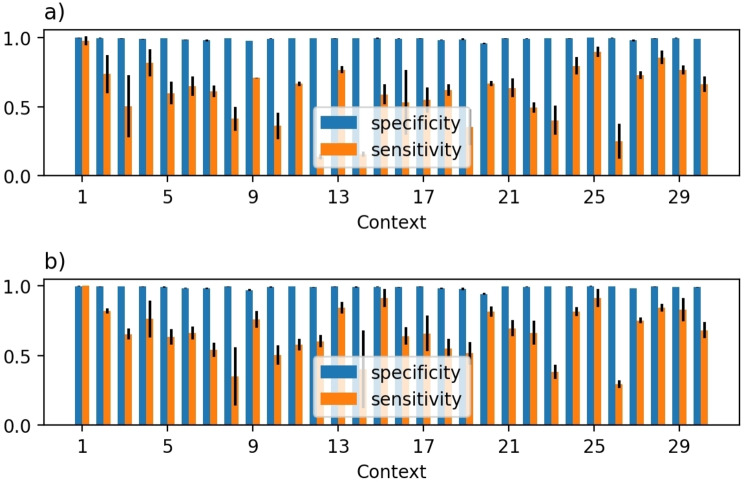
Sensitivity and specificity for all context for a) single‐label model, b) multi‐label model. The error bars indicate one standard deviation over three independent runs.

For both models, the specificity is high, whereas the sensitivity is lower (Figure 6). This pattern suggests that the model retrieves the true positives less well than true negatives. On average, the specificity decreases by 0.002 when going from single‐label to multi‐label model, but the sensitivity increases by 0.07. These both changes are significant at the 95 % confidence interval, with *p*‐values from a paired t‐test being 0.022 for specific and 0.005 for sensitivity. The comparison shows that the single‐label and multi‐label models perform similarly well and on average both predict one context for a reaction.

We did a cross‐comparison for the different contexts by computing the likelihood of predicting a context *x* in top‐1 and top‐3, given that the ground‐truth context is *y* (Figure S2). The sum of the diagonal corresponds to the average top‐1 and top‐3 accuracy. We observe that if the context is common (has a low context index) it will be more often predicted in top‐3 regardless of what the ground‐truth label is and thus more often confused with other contexts. And conversely, if the context is less common (higher context index) it is less often predicted, and more often confused with more common contexts. We also observe that most of the off‐diagonal likelihoods disappears if we look at top‐1 instead of top‐3, indicating that the off‐diagonal predictions stem from top‐2 and top‐3 ranks. These observations hold true for both the single‐label and multi‐label models, in fact there is very little difference between the two models.

**Figure 7:**
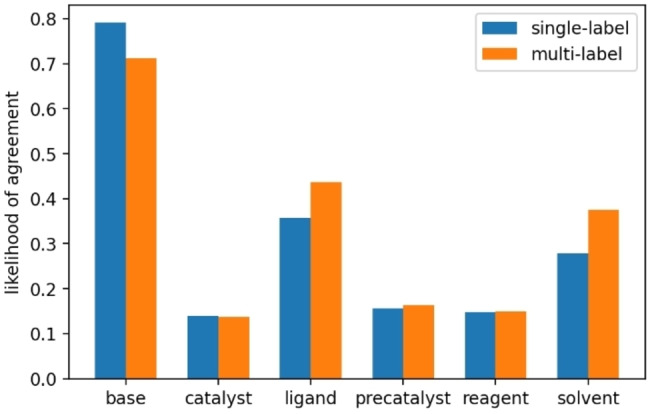
Likelihood agreement between top‐1 prediction and true condition for different condition categories when the true condition is outside top‐3.

**The multi‐label model is better in partially predicting the correct context**. To further outline the difference between the multi‐label and single‐label approaches, we next analyzed the single‐label predictions not in the top‐3 to see how close the top‐1 prediction was to the true context. We assigned a score to each of those predictions, the average number of chemical species that agree when comparing the top‐1 prediction to the true context. The average score is 0.47, indicating that the model predicts that about half of the conditions were correct. In Figure 7, we outline the likelihood of a specific category‘s correctness. The most properly predicted species is the base, which agrees with a likelihood of 0.79, followed by the ligand as 0.36. The catalyst is the most difficult category since this is only correctly predicted in 0.14 of cases. That the base is the easiest to get right follows most likely from that there are only three unique bases in the 30 contexts that the model is built upon. In contrast, the other categories have between six and eight unique compounds.

We also conducted a similar analysis on the multi‐label predictions, for which the highest‐ranked true context was not in the top‐3. For such predictions we compared the top‐1 prediction to the highest‐ranked true context. The average agreement score is 0.50, indicating that the multi‐label prediction is marginally closer to the true context on average. For the ligand and solvent categories, the likelihood of agreement is higher or equal for the multi‐label model. These results illustrate that we can potentially improve the models by exploiting multi‐label data. However, we stress that the contexts predicted herein are completely orthogonal in the sense that the models can never combine, e. g., a catalyst from one context and a ligand from another, although there might be some inherent overlap. If the combination of chemical species were not in the training data, the model cannot predict it, and thus the analysis presented in Figure 7 should be interpreted as that the multi‐label model is marginally more performant in finding a set of conditions with a high overlap with the recorded set of conditions.

**Figure 8:**
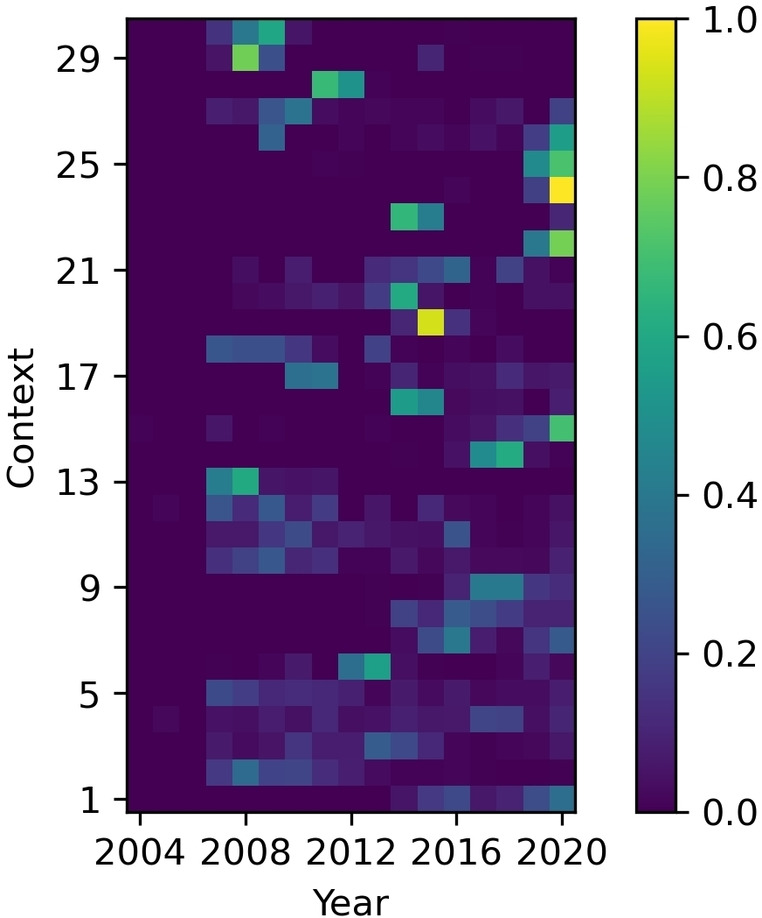
the distribution over time for the top 30 contexts. The brightness (blue ‐ yellow) of the heatmap shows the relative frequency of a context for that year. The contexts are sorted by popularity so that context 1 is the most popular.

**The dataset has strong temporal characteristics**. We analyzed the time‐dependency of context usage by counting the number of times a context has been used for a particular year as shown in Figure 8. Results from this analysis show a temporal effect of context usage. The temporal nature differs between contexts, where some appear consistently throughout time, while others exhibit periods of short popularity. Chemists reporting in the ELNs, use specific contexts for eight years on average, and the average longest range of years a context was used is six. It shows that contexts usage varies from year to year. For instance, the top‐ranking context emerged around 2015, which replaced the context ranked second, used primarily before 2015. Taken together, these observations indicate influence from a variety of different factors, for example, the appearance of new reagents in the scientific literature inciting the adoption of new laboratory strategies or the hiring of new staff having, for instance, different skills or habits. Indeed, there is intense research focus on finding new reagents, especially ligands and catalysts, in the field of the Buchwald‐Hartwig reaction.[^13^, ^14^] Additionally, the internal availability of chemicals such as catalysts and ligands may also guide the context choice in some synthesis projects. The temporal characteristics of the dataset have not been considered in previous modeling studies,[^10^, ^12^] although none have attempted to create a model specifically for Buchwald‐Hartwig reactions. Still, it is likely that the same trends can be observed for other reaction classes.

**Models need to be retrained periodically and historical data is not always useful**. The purpose of the models trained herein is to predict common reaction contexts or to drive an automation platform that leverages contexts optimal for multiple reactions. However, this type of model will often fail to predict the use of new promising reagents, which might be what the chemists principally try first. The model data will have to be updated regularly and ideally reflect the current state of the art to avoid this issue. However, curating such a dataset is non‐trivial as old contexts will often outnumber the new and promising ones. It is interesting to analyze if the model benefits at all from historical data, or if such data should be removed from the training. To investigate these hypotheses with our current data set, we created several single‐label models to predict contexts for reactions recorded in the last two years (2019–2020). We varied what training set to include reactions recorded in different spans of years. We acknowledge that our exemplified data will present a further unbalanced set of labels (in addition to those outlined in the exploration section above), and that test sets may contain labels underrepresented or absent in training. For the aforementioned single‐label model, we re‐calculate the top‐1 and top‐3 accuracies by including only the reactions recorded in 2019 and 2020 (Table 2). The accuracy of this reaction subset is on par with the accuracy of the entire test set, with top‐1 equal to 70 % and top‐3 equal to 0.89 %. We next trained a model on reactions from 2019 to 2020. We observe that this model slightly outperforms the model trained on reactions from all years, with top‐1 accuracy of 72 % and top‐3 accuracy of 92 %, which reflects that data from the same (short) time periods tend to be more consistent and consequently easier to predict. Next, we trained models without including any reactions from 2019 and 2020 and investigated the predictivity on contexts for those years. Understandably, performance drops when excluding data for those years from modeling, reflecting that historical data are, as expected, not sufficient to predict more recent experiments. Further, a model trained on reactions between 2014 and 2018 had a top‐1 accuracy of only 19 % and top‐3 accuracy of 33 %, whilst performance increases for a model trained between 2015–2018, with top‐1 accuracy of 32 % and top‐3 accuracy of 54 %. Taken together, this shows that predictions are more accurate when the training is temporally close to the test data.

**Table 2:** Accuracy of prediction contexts for reactions recorded in 2019 and 2020 for models trained on various ranges of years.

Training set	top‐1 accuracy	top‐3 accuracy
2004–2020	0.70±0.02	0.89±0.01
2019–2020	0.72±0.01	0.92±0.01
2004–2018	0.19±0.01	0.33±0.03
2015–2018	0.32±0.01	0.54±0.01

The outcomes of the simple temporal validation experiments presented in this section highlight the need for periodical retraining of the models such as those described herein, to ensure their usefulness for medicinal chemistry projects. None of the previously published studies for context prediction performed a temporal analysis,[^10^, ^12^] which indicates that they may be prone to similar deficiencies. While we highlight the limitations of only relying on historical data here, it also is important to stress that historical data might still be chemically relevant, and an old context could be just as effective as a new one. Indeed, most ligands can yield high product concentrations in cases where the amine and halide are highly suitable for this type of reaction. Thus, it is not always needed to employ the more recent and supposedly more performant contexts for all planned synthesis, although as it is clear from Figure 8 that chemists tend to use more recent contexts.

## 4 Conclusions

We have introduced novel models to predict the chemical context for Buchwald‐Hartwig coupling reactions. In particular, our models predict the chemical conditions jointly, i. e., given a query reaction, it predicts a set of ligand, base, solvent, and (pre‐)catalyst. We show that both single‐label and multi‐label models predict the chemical context well, reaching a top‐3 accuracy above 90 %. The model trained on multi‐label data has better sensitivity on individual contexts and is better at predicting parts of the context. These findings show that there is a possibility of training a prediction model for a chemical context and that there is some advantage of including multi‐label data. Although to fully explore the relative advantage of a multi‐label model, another, more multi‐labeled, dataset is needed. Overall, the current models can be used to predict the context of Buchwald‐Hartwig reactions from a limited set of contexts, for example, in the scenario of an automated platform requiring a set of well‐known conditions. However, as shown by the time‐dependency analysis, such models most likely need to be updated regularly to encompass changed commercial availability and novel science. This modeling requirement has been neglected in previous modeling studies[^10^, ^12^] and thus brings the relevancy of such models into question. We have clearly shown that, at least for Buchwald‐Hartwig reactions, we cannot fully exploit historical data to predict more recent contexts, and it is likely that this is true for other reaction classes. On the other hand, from a chemical perspective, models based on historical data can be useful in some contexts, and older contexts might be as chemically effective as newer ones. To assess if alternative contexts predicted by the models can work in the laboratory is outside the scope of a modelling study like this, and can anyway only be truly evaluated with extensive experimentation. A potential avenue for future research is to augment the data driven‐approach used herein with physics‐based descriptors such as reactivity to develop models that can provide other types of recommendations. Nevertheless, we believe that the findings presented herein will be a template for creating models for other types of reactions. Although extra attention to the data and periodic re‐trained is required, we envisage that such models will be useful in future synthesis planning.

## Author Contributions

S.G., O.E., S.O, T.K. conceptualized research. S.G, A.M., G.L, and T.K. carried out research. S.G., S.O. and T.K supervised research, analyzed results, and wrote manuscript. All authors edited manuscript drafts.

## Conflict of interest

None declared.

5

## Supporting information

As a service to our authors and readers, this journal provides supporting information supplied by the authors. Such materials are peer reviewed and may be re‐organized for online delivery, but are not copy‐edited or typeset. Technical support issues arising from supporting information (other than missing files) should be addressed to the authors.

Supporting Information

## Data Availability

The data used in this study is proprietary and is thus not available. The code used to perform the model building is available at https://doi.org/10.5281/zenodo.5599325.
