# Supplementary material for: Prediction of the Chemical Context for Buchwald‐Hartwig Coupling Reactions
Source: Mol Inform. 2022 Feb 22;41(8):2100294. doi: 10.1002/minf.202100294 (PMC9540548; doi:10.1002/minf.202100294)
Supplement: Supplementary file 1 — Supporting Information [file MINF-41-2100294-s001.pdf]

molecular informatics

Supporting Information

Prediction of the Chemical Context for Buchwald-Hartwig Coupling Reactions

Samuel Genheden,* Agnes Mårdh, Gustav Lahti, Ola Engkvist, Simon Olsson,* and Thierry Kogej© 2022 The Authors. Molecular Informatics published by Wiley-VCH GmbH. This is an open access article under the terms of the Creative Commons Attribution License, which permits use, distribution and reproduction in any medium, provided the original work is properly cited.

Supporting Information:

Prediction of the chemical context for Buchwald-Hartwig coupling reactions

Samuel Genheden, Agnes Mårdh, Gustav Lahti, Ola Engkvist, Simon Olsson, Thierry Kogej

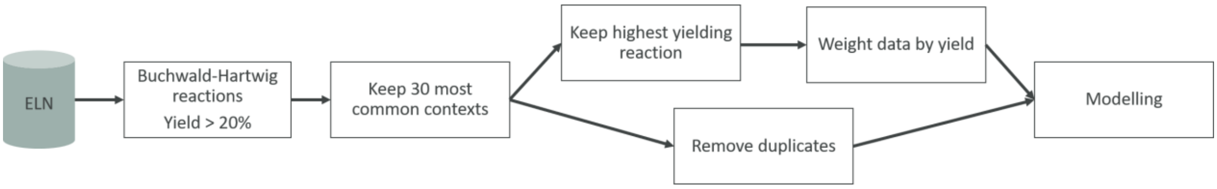

Figure S1 – Overview of the data-processing pipeline to arrive at modelling-ready data.

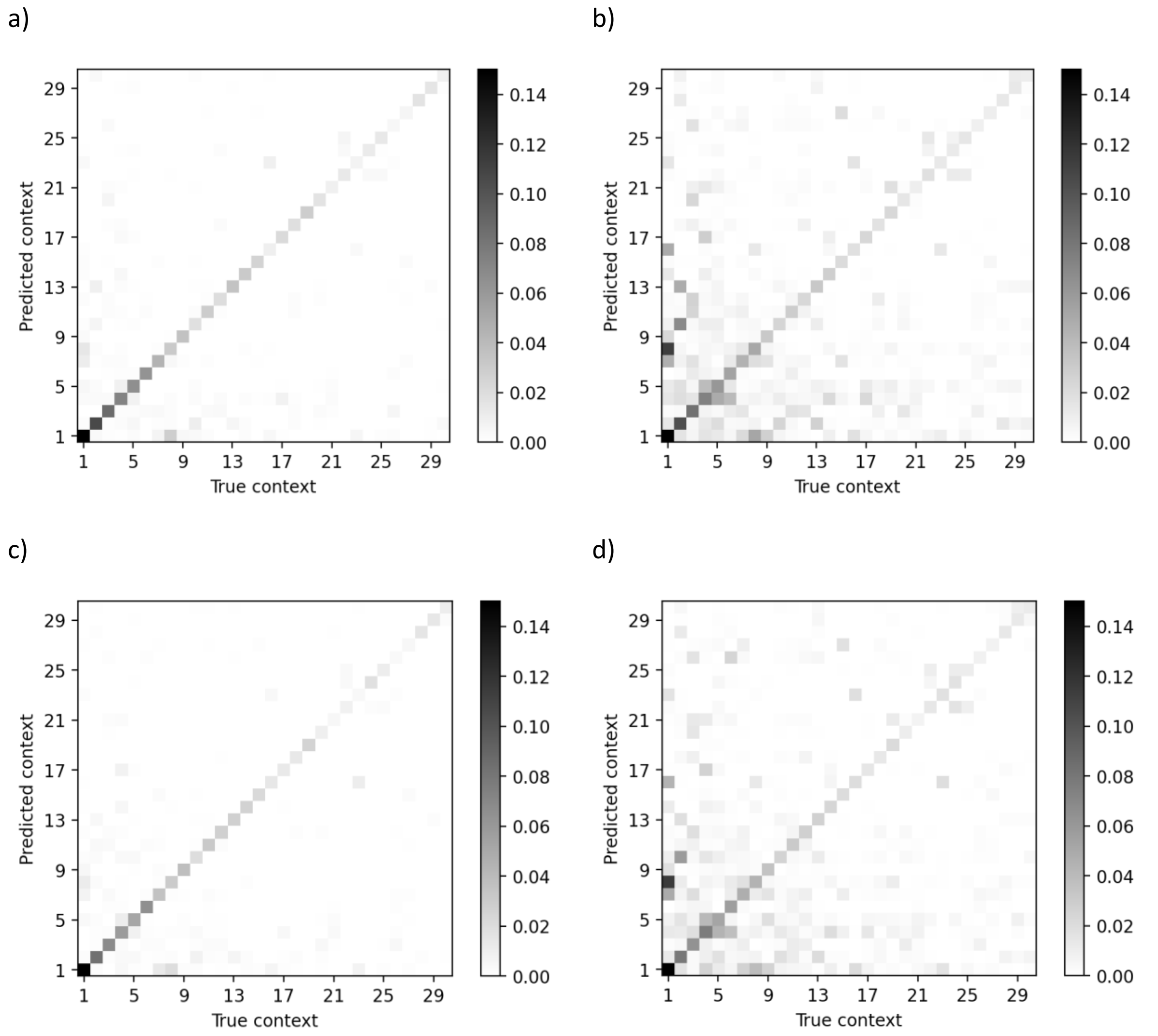

Figure S2 – Likelihood of predicting a context *x* when the ground-truth context is *y*. a) Single-label predictions in top-1, b) Single-label predictions in top-3, c) Multi-label predictions in top-1, d) Multi-label predictions in top-3. The likelihood is normalized based on the total number of data points and is computed over all three trained models. The contexts are sorted based on popularity in the full dataset, where 1 is the most common context.
